# Supplementary material for: A metabolic profile of all-cause mortality risk identified in an observational study of 44,168 individuals
Source: Nat Commun. 2019 Aug 20;10:3346. doi: 10.1038/s41467-019-11311-9 (PMC6702196; doi:10.1038/s41467-019-11311-9)
Supplement: Supplementary file 1 — Supplementary Information [file 41467_2019_11311_MOESM1_ESM.pdf]

A metabolic biomarker profile of all-cause mortality risk identified in an observational study of 44,168 individuals.

Deelen *et al*.

Table of contents **Pages**

1.	Supplementary Methods	3-5
2.	Supplementary Note 1	6-7
3.	Supplementary Figures	8-26
4.	Supplementary Tables	27-30
5.	Supplementary References	31

Supplementary Methods

Study populations

Alpha Omega Cohort
The Alpha Omega Cohort consists of 4,837 Dutch men and women aged 60-80 years with a clinically diagnosed myocardial infarction <10 years before study enrollment. Baseline examinations and blood sampling took place between 2002 and 2006, after which the cohort has been followed up for cause-specific mortality. During the first 3 years of follow-up, patients participated in an intervention study with omega-3 fatty acids (Alpha Omega Trial).[1, 2] For the present analysis, a random sample of 600 patients was selected, who were followed up through Statistics Netherlands (CBS) until the 1st of January 2014. Metabolic biomarkers were successfully quantified in EDTA plasma samples of 568 patients. The Alpha Omega Cohort is registered with clinicaltrials.gov (Identifier: NCT03192410).

Avon Longitudinal Study of Parents and Children (ALSPAC)
The Avon Longitudinal Study of Parents and Children (ALSPAC) recruited pregnant women resident in and around the City of Bristol, UK, with expected dates of delivery from 1st April 1991 to 31st December 1992. Women carrying 14,541 pregnancies were initially recruited from a total eligible sample of 20,248 eligible pregnancies and provided a wide range of data (see ALSPAC Data Dictionary: http://www.bris.ac.uk/alspac/researchers/data-access/data-dictionary/).[3] Ethical approval for the study was obtained from the ALSPAC Ethics and Law Committee and the Local Research Ethics Committees. Mortality data were sourced from record linkage to the UK mortality register (managed by the National Health Service and the Office of National Statistics (ONS). Due to the terms of our data sharing agreement with the ONS, ALSPAC does not permit the sharing of mortality records beyond a small group of 'ONS Approved Researcher' staff. These staff are directly employed by ALSPAC and operate within a secure research setting (developed using Data Safe Haven principles, see Burton et al.).[4] To facilitate this investigation, synthetic mortality data was created and used to develop analytical syntax, which in turn was run using the real data within the ALSPAC secure environment. Aggregate outcomes were checked for disclosure risk prior to circulation to other authors and inclusion in this manuscript. Metabolic biomarkers were successfully quantified in fasting serum samples collected from 4,531 mothers.

Erasmus Rucphen Family (ERF) study
The Erasmus Rucphen Family (ERF) study is a family-based study that includes inhabitants of a genetically isolated community in the South-West of the Netherlands, ascertained as part of the Genetic Research in Isolated Population program.[5] The ERF cohort includes approximately 3,000 living descendants of 22 founder couples who had at least 6 children baptized in the community church. Individuals who were 18 years or older were invited to participate in the study[5]. Baseline data were collected between 2002 and 2005 and follow-up data between 2015 and 2018. In total, 680 individuals were included in this study. Metabolic biomarkers were successfully quantified in fasting EDTA plasma samples for all individuals. All-cause mortality information was available until the 25th of June 2015. The ERF study was approved by the medical ethics committee of the Erasmus Medical Center, Rotterdam, The Netherlands. All participants provided written informed consents and all investigations were carried out in accordance with the Declaration of Helsinki.

Estonian Biobank cohort (EGCUT)
Estonian Biobank (Estonian Genome Center, University of Tartu, http://www.biobank.ee) is a population-based biobank that recruited a cohort of 51,830 participants, including adults (minimum age 18 years) from all counties in Estonia and accounting for approximately 5% of the Estonian adult population during the main recruitment period (2002-2011). At baseline, an extensive phenotype questionnaire was conducted together with a measurement panel. Follow-up data for the cohort is available from linkage with national health-related registries and the Estonian Health Insurance database.[6] The data on cause-specific registry is obtained from the linkage of the cohort database with the Estonian Causes of Death Registry (http://www.tai.ee/en/r-and-d/registers/estonian-causes-of-death-registry). Metabolic biomarkers were successfully quantified in non-fasted plasma samples collected from a random subset of 10,988 individuals from the cohort.

FINRISK 1997 cohort + Dietary, Lifestyle and Genetic Predictors of Obesity and Metabolic Syndrome (DILGOM) study
The National FINRISK study is a Finnish population-based chronic disease risk factor monitoring survey carried out at 5-year intervals since 1972.[7] For each survey, a representative random sample was selected from 25- to 74-year-old inhabitants of different regions in Finland. The survey included a questionnaire and a clinical examination, at which a blood sample was drawn, with linkage to national registers of cardiovascular and other health outcomes. This study included all participants available from FINRISK 1997 clinical examination. For

FINRISK 2007, a total of 6,258 subjects participated and returned the questionnaire. All FINRISK 2007 participants were invited to the Dietary, Lifestyle and Genetic Predictors of Obesity and Metabolic Syndrome (DILGOM) study that was used here.[8] Of the invited, 5,024 subjects (participation rate: 84%) participated in the DILGOM study visit in April–June 2007. During the study visit subjects completed a FFQ and other questionnaires on health-related behavior. Fasting blood samples were drawn as a part of DILGOM visit. Metabolic biomarkers were successfully quantified in 7,603 individuals from FINRISK 1997 and 4,816 from DILGOM.

Cooperative Health Research in the Region of Augsburg (KORA) study

Cooperative Health Research in the Region of Augsburg (KORA) is a research platform for population-based surveys and follow-up studies of adult residents in the city of Augsburg and the two adjacent counties in Southern Germany.[9] In the present work, we used data from the KORA F4 study, which was conducted between 2006 and 2008 as a follow-up of the fourth KORA survey (KORA S4). 3,080 of the 4,261 subjects who were recruited between 1999 and 2001 (S4) at the age of 25-74 years participated in the KORA F4 follow-up. These subjects were further followed up for all-cause mortality until 2015. For a total of 1,790 participants, metabolic biomarkers were quantified in fasting serum samples drawn at the KORA F4 visit.

Leiden Longevity Study (LLS)

The Leiden Longevity Study (LLS) consists of 421 long-lived families of European descent. Families were included if at least two long-lived siblings were alive and fulfilled the age criterion of 89 years or older for males and 91 years or older for females, representing <0.5% of the Dutch population in 2001.[10] In total, 944 long-lived proband siblings (mean age = 94 years, range = 89-104), 1,671 offspring (mean age = 61 years, range = 39-81) and 744 spouses thereof (mean age = 60 years, range = 36-79) were included. Registry-based follow-up until the 27th of October 2016 was available for all participants. Metabolic biomarkers were successfully quantified in 843 nonagenarians, 1,157 of their offspring and 684 controls using non-fasted EDTA plasma samples.

PROspective Study of Pravastatin in the Elderly at Risk (PROSPER)

The PROspective Study of Pravastatin in the Elderly at Risk (PROSPER) trial design has been published.[11] In brief, 5,804 elderly adults (70-82 years old) were enrolled. This was a double-blind, randomised placebo controlled trial investigating the benefit of pravastatin (40 mg/day) in elderly individuals at risk of CVD. Participants were identified in the primary care setting from 3 centres: Glasgow, Scotland; Cork, Ireland or Leiden, the Netherlands. All participants had high-normal to high cholesterol (4.0-9.0 mmol/L) at baseline. Additionally 50% of patients had evidence of vascular disease (physician diagnosed stable angina, stroke, transient ischaemic attack (TIA) or myocardial infarction (MI)) and the remaining 50% of patients had high risk of vascular disease as they had either hypertension, diabetes or were smokers. The primary outcome measure of PROSPER was a composite CVD outcome. In the current study the endpoint of interest was all-cause mortality. Patients were recruited between December 1997 and May 1999 and the mean follow-up period was 3.2 years. Fasting venous blood samples were collected at baseline and at 3-month intervals and biobanked at -80°C. For the present study previously unthawed 6-month post-randomisation samples were used, employing the study as a cohort study and adjusting for randomised treatment in the analyses. Metabolic biomarkers were successfully quantified in 5,329 individuals.

Rotterdam Study

The Rotterdam Study (RS) is a prospective population-based study designed to investigate the determinants of disease occurrence and progression in the elderly.[12] The RS cohort was initially defined in 1990 among 7,983 persons living in the well-defined Ommoord district in Rotterdam, The Netherlands. All participants underwent a home interview and extensive physical examination at baseline and during follow-up examinations occurring every 3–4 years (RS-I). The cohort was further extended in 2000 (RS-II) and 2006 (RS-III), establishing a total of 14,926 participants aged 45 years or over.[12] In total, 2,963 individuals were included in this study. Metabolic biomarkers were successfully quantified in fasting EDTA plasma samples for all individuals. All-cause mortality information was available until the 5th of March 2015. The RS has been approved by the Medical Ethics Committee of the Erasmus MC (registration number MEC 02.1015) and by the Dutch Ministry of Health, Welfare and Sport (Population Screening Act WBO, license number 1071272-159521-PG). The RS has been entered into the Netherlands National Trial Register (NTR; www.trialregister.nl) and into the WHO International Clinical Trials Registry Platform (ICTRP; www.who.int/ictrp/network/primary/en/) under shared catalogue number NTR6831. All participants provided written informed consent to participate in the study and to have their information obtained from treating physicians.

TwinsUK
TwinsUK is a UK register of 12,000 twins. Participants are continuously recruited from the general population since 1992 through media campaigns.[13] For this study, 1,996 individuals (mean age 64.58± 8.53) were used in which metabolic biomarkers were successfully quantified using fasted serum samples.

Supplementary Note 1

Acknowledgements

Alpha Omega Cohort
Financial support for the Alpha Omega Cohort was obtained from the Dutch Heart Foundation (grant 200T401) and the National Institutes of Health (grant R01HL076200), USA. DNA isolation was funded by BBMRI-NL (grant CP2011-18). E. Waterham is acknowledged for data management and governance of the biobank of the Alpha Omega Cohort.

Avon Longitudinal Study of Parents and Children (ALSPAC)
We are extremely grateful to all the families who took part in this study, the midwives for their help in recruiting them, and the whole ALSPAC team, which includes interviewers, computer and laboratory technicians, clerical workers, research scientists, volunteers, managers, receptionists and nurses. The UK Medical Research Council and the Wellcome Trust (Grant ref: 102215/2/13/2) and the University of Bristol provide core support for ALSPAC. A. Boyd is funded by the Wellcome Trust (Grant ref: WT086118/Z/08/Z). G. Davey Smith and F. Drenos are supported by the University of Bristol and the UK Medical Research Council (MC_UU_12013/1–9). The National Institute for Health Research (NIHR) NF-SI-0611-10196 funded the ALSPAC mother's metabolomics data.

Erasmus Rucphen Family (ERF) study
The Erasmus Rucphen Family (ERF) study has received funding from the Centre for Medical Systems Biology (CMSB) and Netherlands Consortium for Systems Biology (NCSB), both within the framework of the Netherlands Genomics Initiative (NGI)/Netherlands Organization for Scientific Research (NWO). ERF study is also a part of EUROSPAN (European Special Populations Research Network) (FP6 STREP grant number 18947 (LSHG-CT-2006-018947)); European Network of Genomic and Genetic Epidemiology (ENGAGE) from the European Community's Seventh Framework Programme (FP7/2007-2013)/grant agreement HEALTH-F4-2007-201413; "Quality of Life and Management of the Living Resources" of 5th Framework Programme (no. QLG2-CT-2002-01254); FP7 project EUROHEADPAIN (nr 602633), the Internationale Stichting Alzheimer Onderzoek (ISAO); the Hersenstichting Nederland (HSN); and the JNPD under the project PERADES (grant number 733051021, Defining Genetic, Polygenic and Environmental Risk for Alzheimer’s Disease using multiple powerful cohorts, focused Epigenetics and Stem cell metabolomics). Metabolomics measurements of ERF has been funded by Biobanking and Biomolecular Resources Research Infrastructure (BBMRI)-NL (184.021.007). The ERF-follow up study is funded by CardioVasculair Onderzoek Nederland (CVON 2012-03). We are grateful to all study participants and their relatives, general practitioners and neurologists for their contributions and to P. Veraart for her help in genealogy, J. Vergeer for the supervision of the laboratory work, both S.J. van der Lee and A. van der Spek for collection of the follow-up data and P. Snijders for his help in data collection of both baseline and follow-up data.

Estonian Biobank cohort
The Estonian Biobank cohort has received funding from the Estonian Research Council Grant IUT20-60, EU H2020 grant 692145, and European Union through the European Regional Development Fund (Project No. 2014-2020.4.01.15-0012) GENTRANSMED. The work of K. Fischer was also supported by the Estonian Research Council grant PUT1665.

FINRISK 1997 cohort + Dietary, Lifestyle and Genetic Predictors of Obesity and Metabolic Syndrome (DILGOM) study
FINRISK 1997 has been mainly funded by the budgetary funds of the National Institute for Health and Welfare. Important additional funding has been obtained from the Academy of Finland, Finnish Foundation for Cardiovascular Research and other domestic foundations. The NMR metabolomics determinations were funded by a grant from the Academy of Finland (#139635 to VS). DILGOM 2007 baseline survey was funded by the Academy of Finland (grant # 136895 and 263836). The study was further supported by Academy of Finland (grant # 283045, 297338 and 307247) and Novo Nordisk Foundation (NNF17OC0026062). V. Salomaa was also supported by the Finnish Foundation for Cardiovascular Research.

Cooperative Health Research in the Region of Augsburg (KORA) study
The KORA study was initiated and financed by the Helmholtz Zentrum München–German Research Center for Environmental Health, which is funded by the German Federal Ministry of Education and Research (BMBF) and by the State of Bavaria. Furthermore, KORA research was supported within the Munich Center of Health Sciences (MC-Health), Ludwig-Maximilians-Universität, as part of LMUinnovativ.

Leiden Longevity Study (LLS)
The LLS has received funding from the European Union's Seventh Framework Programme (FP7/2007-2011) under grant agreement n° 259679. This study was supported by a grant from the Innovation-Oriented Research Program on Genomics (SenterNovem IGE05007), the Centre for Medical Systems Biology, and the Netherlands Consortium for Healthy Ageing (grants 05040202 and 050-060-810), all in the framework of the Netherlands Genomics Initiative, Netherlands Organization for Scientific Research (NWO), Unilever Colworth, and by BBMRI-NL, a Research Infrastructure financed by the Dutch government (NWO 184.021.007). J. Deelen was financially supported by the Alexander von Humboldt Foundation.

PROspective Study of Pravastatin in the Elderly at Risk (PROSPER)
PROSPER was supported by the European Federation of Pharmaceutical Industries Associations (EFPIA), Innovative Medicines Initiative Joint Undertaking, European Medical Information Framework (EMIF) grant number 115372 and the European Commission under the Health Cooperation Work Programme of the 7th Framework Programme (Grant number 305507) "Heart 'omics' in AGEing" (HOMAGE).

Rotterdam Study
The Rotterdam Study is supported by the Erasmus MC University Medical Center and Erasmus University Rotterdam; The Netherlands Organisation for Scientific Research (NWO); The Netherlands Organisation for Health Research and Development (ZonMw); the Research Institute for Diseases in the Elderly (RIDE); The Netherlands Genomics Initiative (NGI); the Ministry of Education, Culture and Science; the Ministry of Health, Welfare and Sports; the European Commission (DG XII); and the Municipality of Rotterdam. The authors are grateful to the study participants, the staff from the Rotterdam Study and the participating general practitioners and pharmacists. Metabolomics measurements were funded by Biobanking and Biomolecular Resources Research Infrastructure (BBMRI)–NL (184.021.007) and the JNPD under the project PERADES (grant number 733051021, Defining Genetic, Polygenic and Environmental Risk for Alzheimer's Disease using multiple powerful cohorts, focused Epigenetics and Stem cell metabolomics).

TwinsUK
TwinsUK was funded by the Wellcome Trust; European Community's Seventh Framework Programme (FP7/2007-2013). The study also receives support from the National Institute for Health Research (NIHR)-funded BioResource, Clinical Research Facility and Biomedical Research Centre based at Guy's and St Tomas' NHS Foundation Trust in partnership with King's College London. T.D. Spector is an ERC Advanced Researcher, C. Menni is funded by the MRC AimHy (MR/M016560/1) project grant.

M. Ala-Korpela is supported by a Senior Research Fellowship from the National Health and Medical Research Council (NHMRC) of Australia (APP1158958). He also works in a unit that is supported by the University of Bristol and UK Medical Research Council (MC_UU_12013/1) and holds a grant from the Sigrid Juselius Foundation. The Baker Institute is supported in part by the Victorian Government's Operational Infrastructure Support Program.

Supplementary Figures

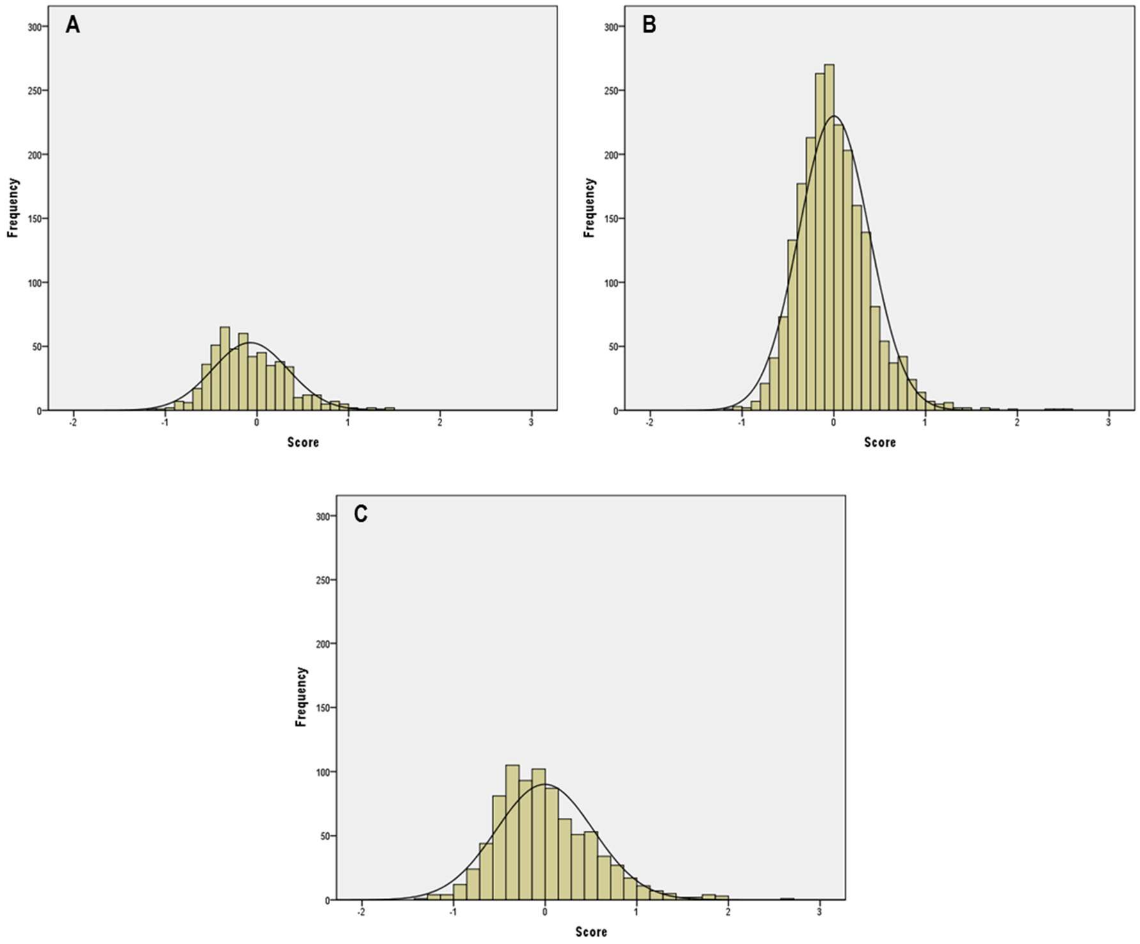

Supplementary Figure 1. Study-specific examples of the distribution of the metabolic biomarker score. Histograms depicting the distribution of the score in the Alpha Omega Cohort (A), Leiden Longevity Study (LLS) offspring and partners (B) and LLS nonagenarians (C).

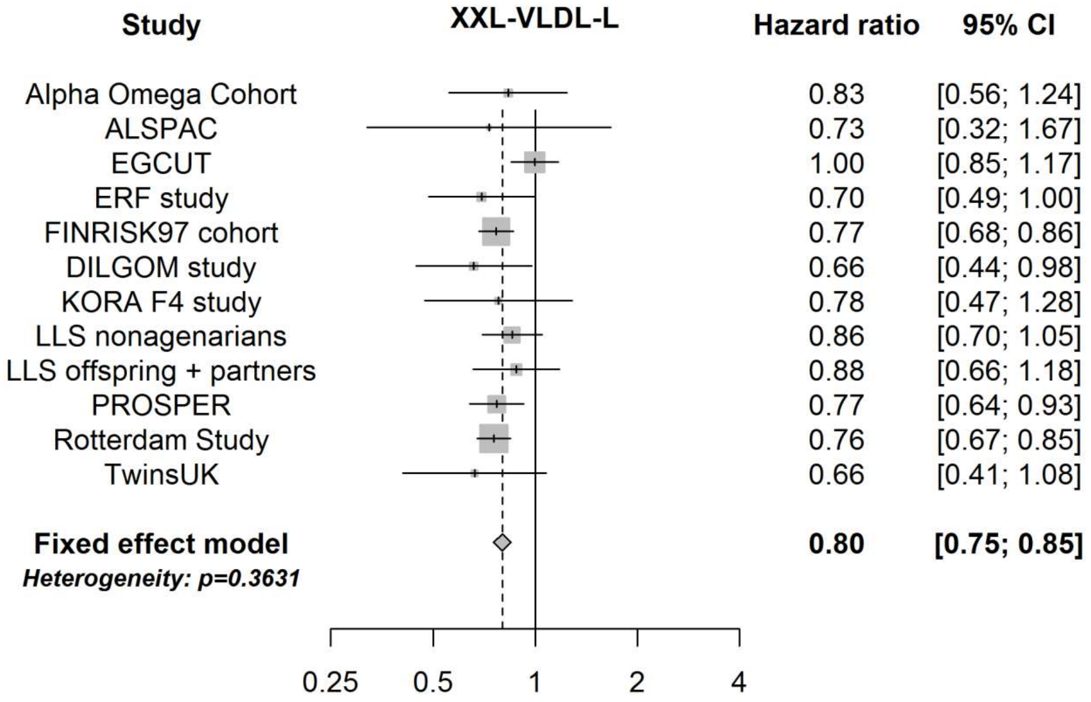

Supplementary Figure 2. Forest plot for the total lipids in chylomicrons and extremely large very low density lipoprotein based on the fully adjusted model.

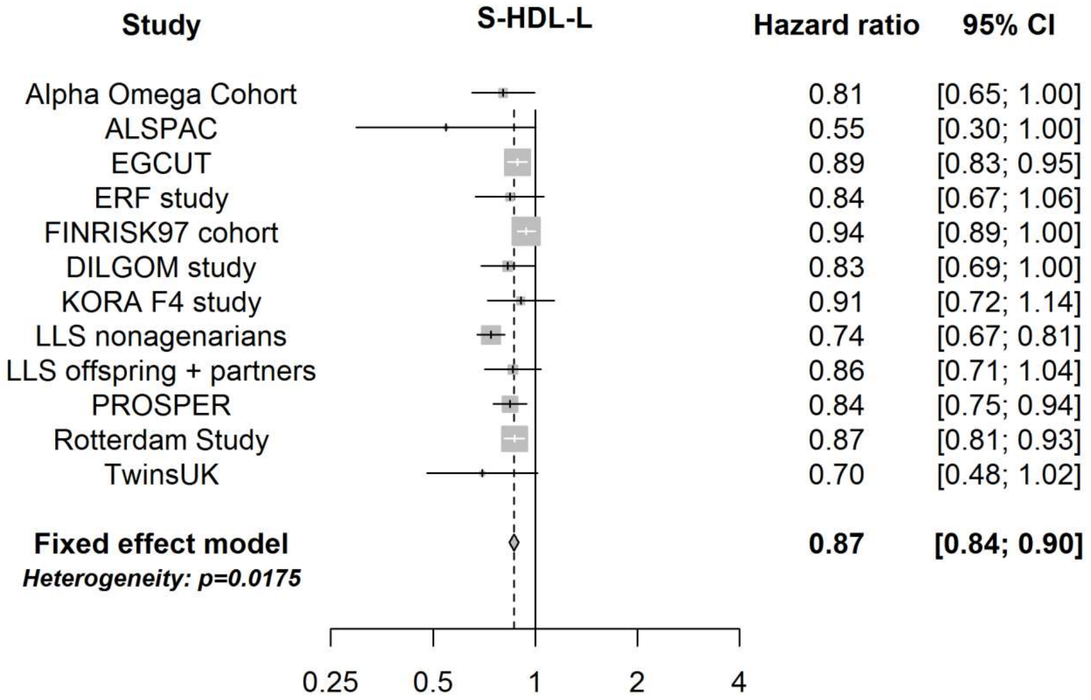

Supplementary Figure 3. Forest plot for the total lipids in small high-density lipoprotein based on the fully adjusted model.

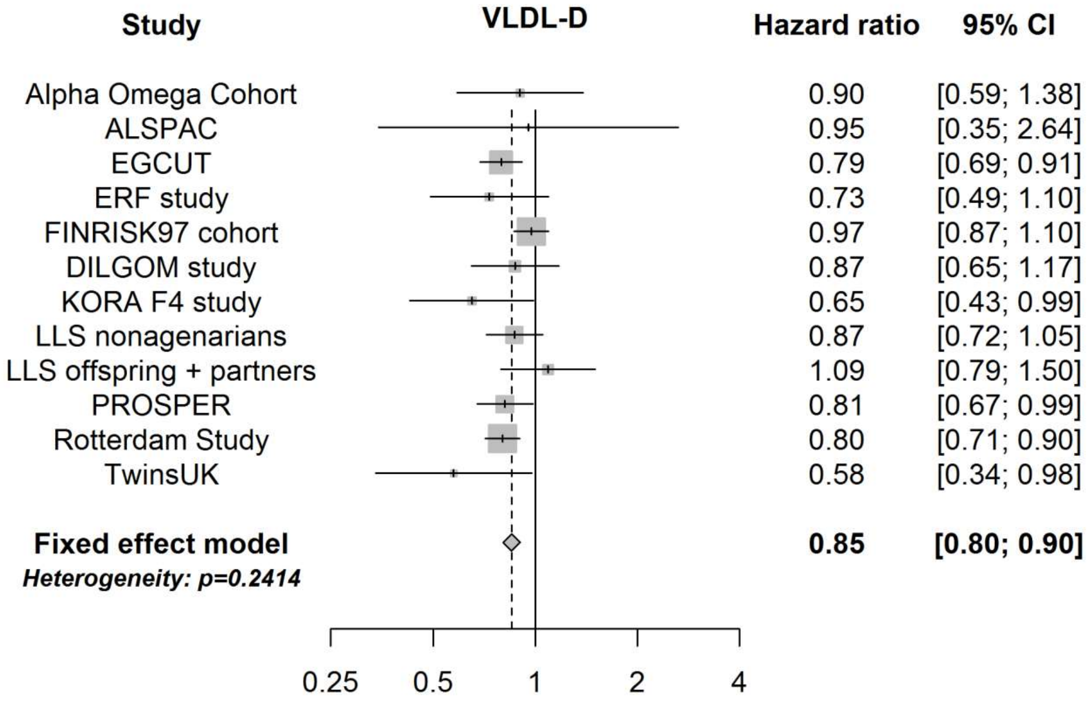

Supplementary Figure 4. Forest plot for the mean diameter for very low density lipoprotein based on the fully adjusted model.

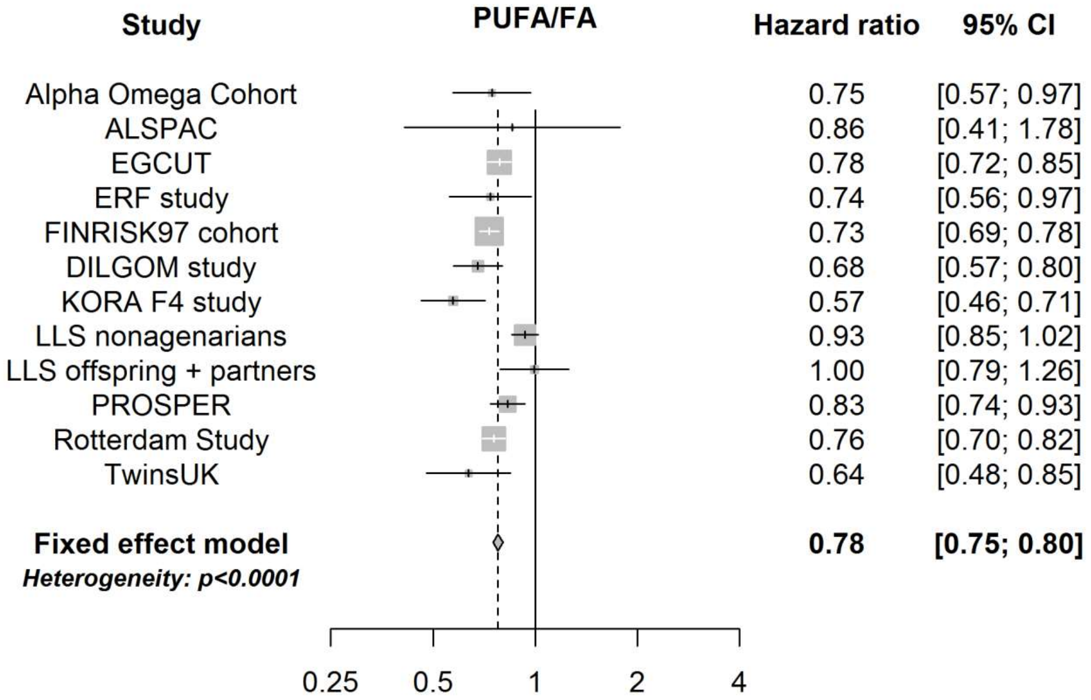

Supplementary Figure 5. Forest plot for the ratio of polyunsaturated fatty acids to total fatty acids based on the fully adjusted model.

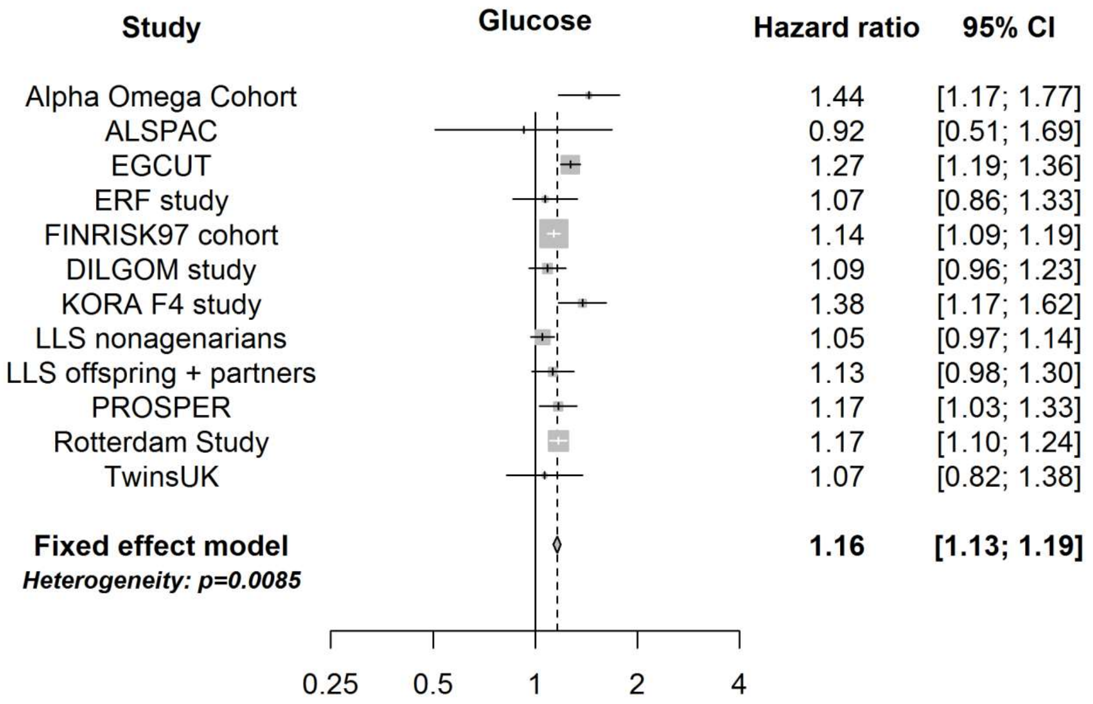

Supplementary Figure 6. Forest plot for glucose based on the fully adjusted model.

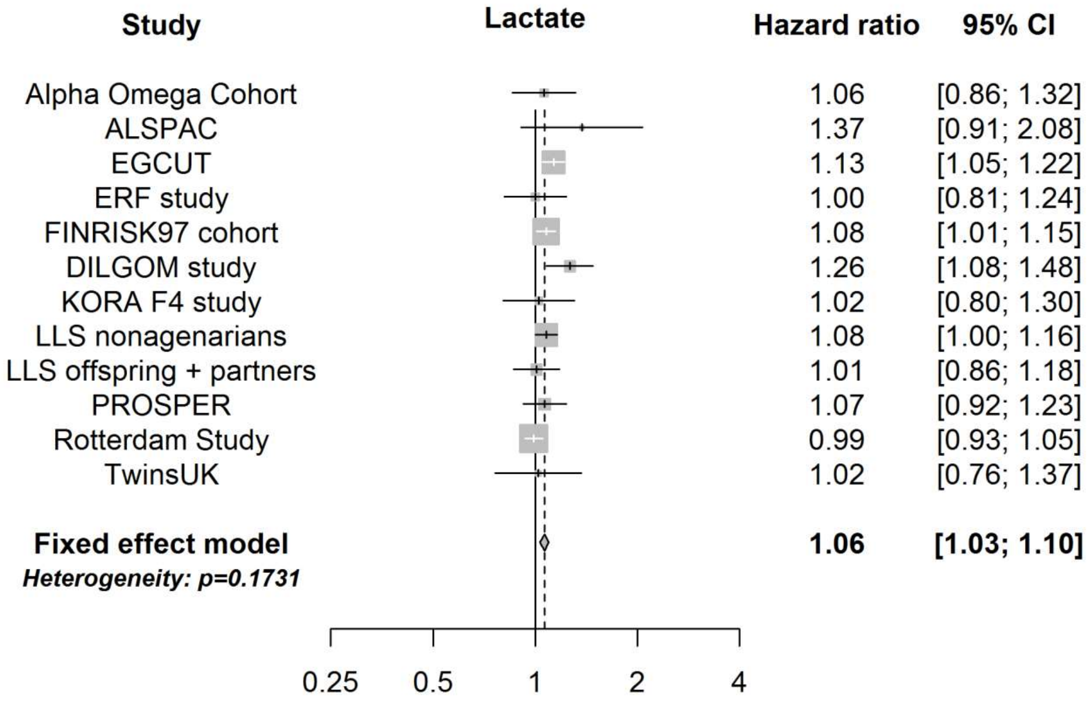

Supplementary Figure 7. Forest plot for lactate based on the fully adjusted model.

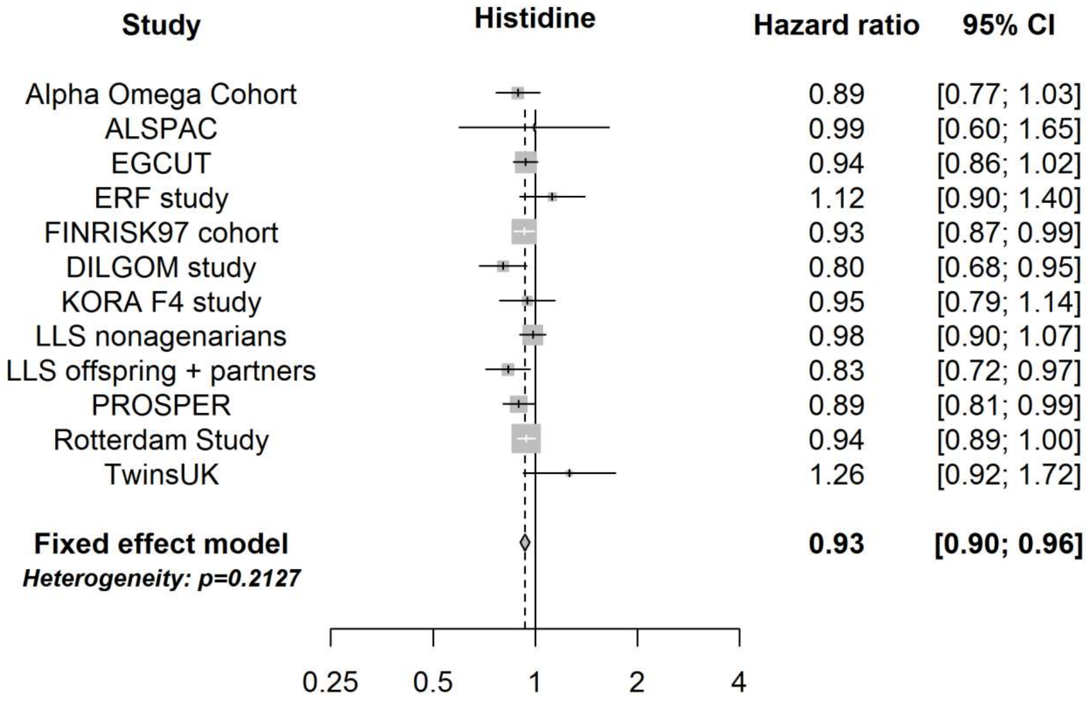

Supplementary Figure 8. Forest plot for histidine based on the fully adjusted model.

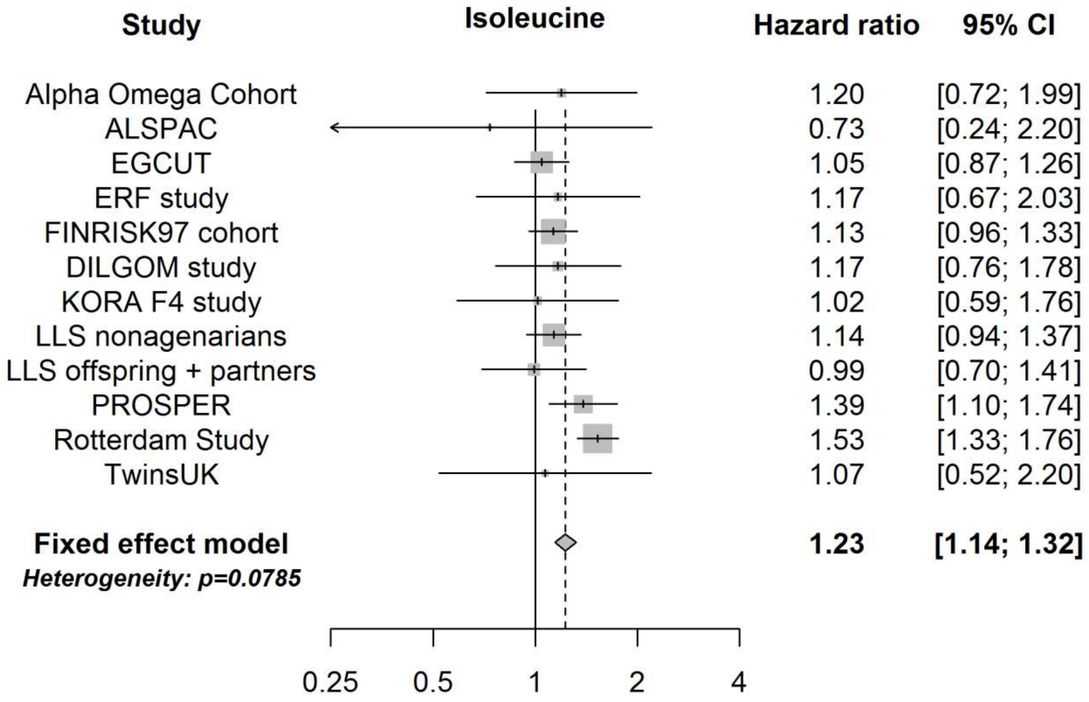

Supplementary Figure 9. Forest plot for isoleucine based on the fully adjusted model.

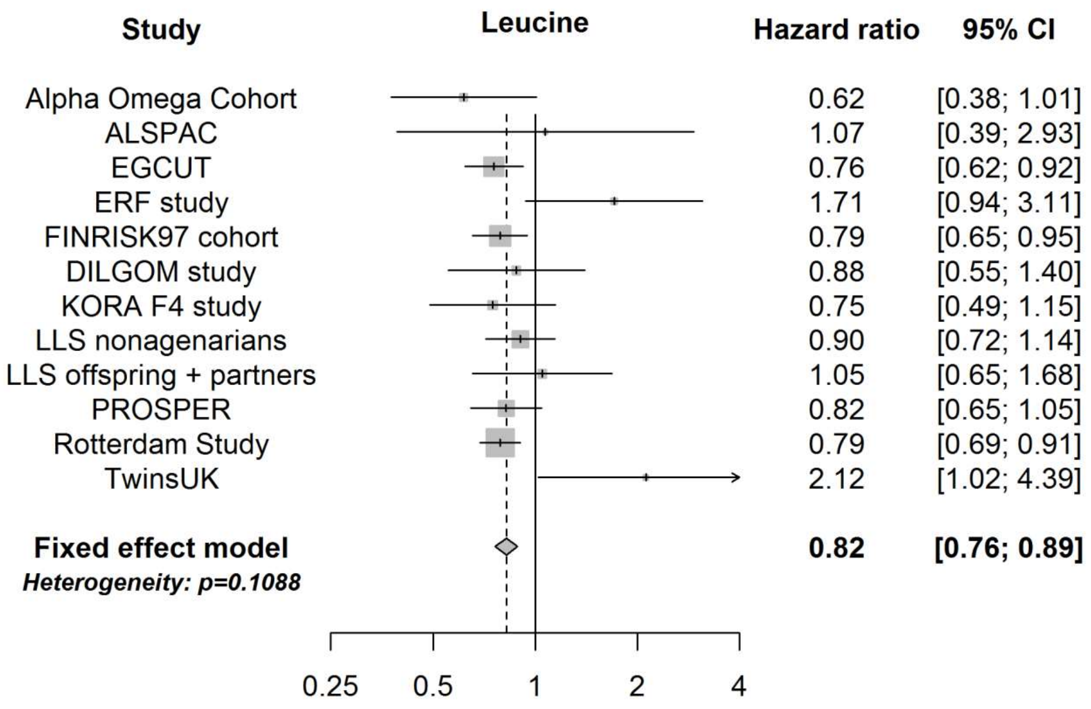

Supplementary Figure 10. Forest plot for leucine based on the fully adjusted model.

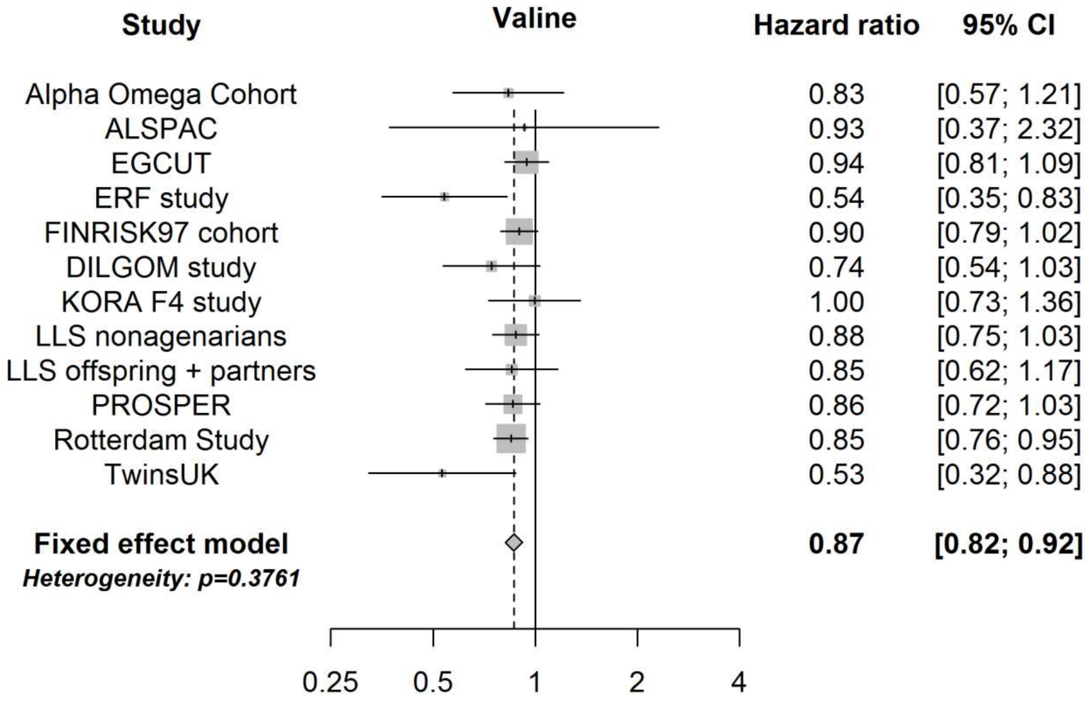

Supplementary Figure 11. Forest plot for valine based on the fully adjusted model.

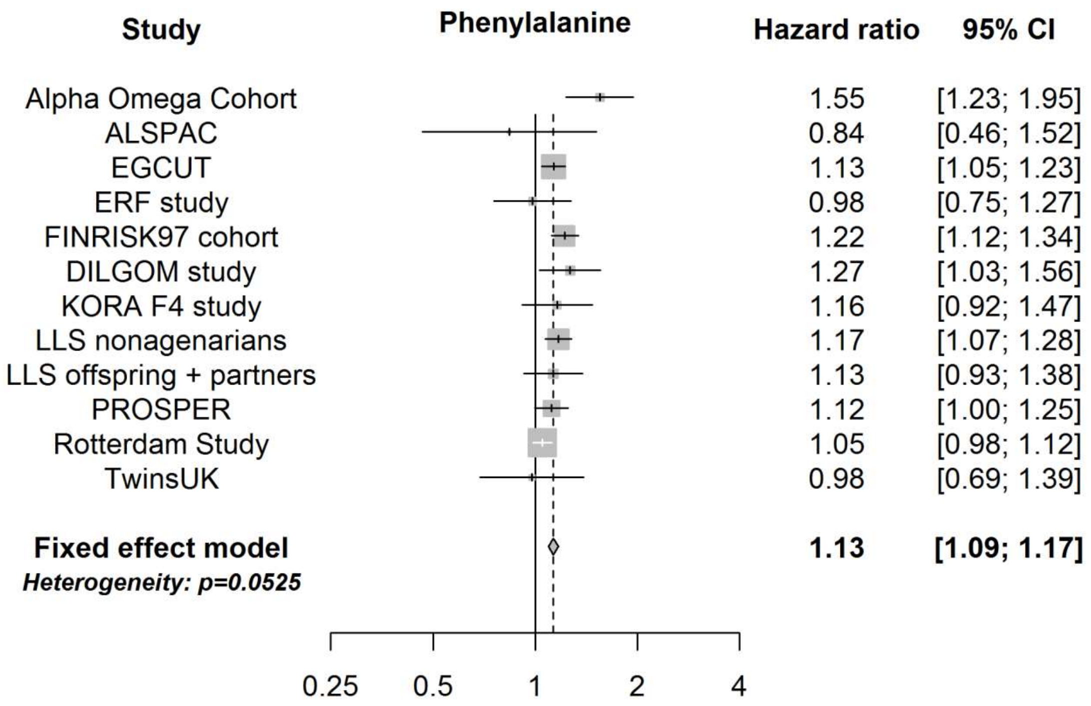

Supplementary Figure 12. Forest plot for phenylalanine based on the fully adjusted model.

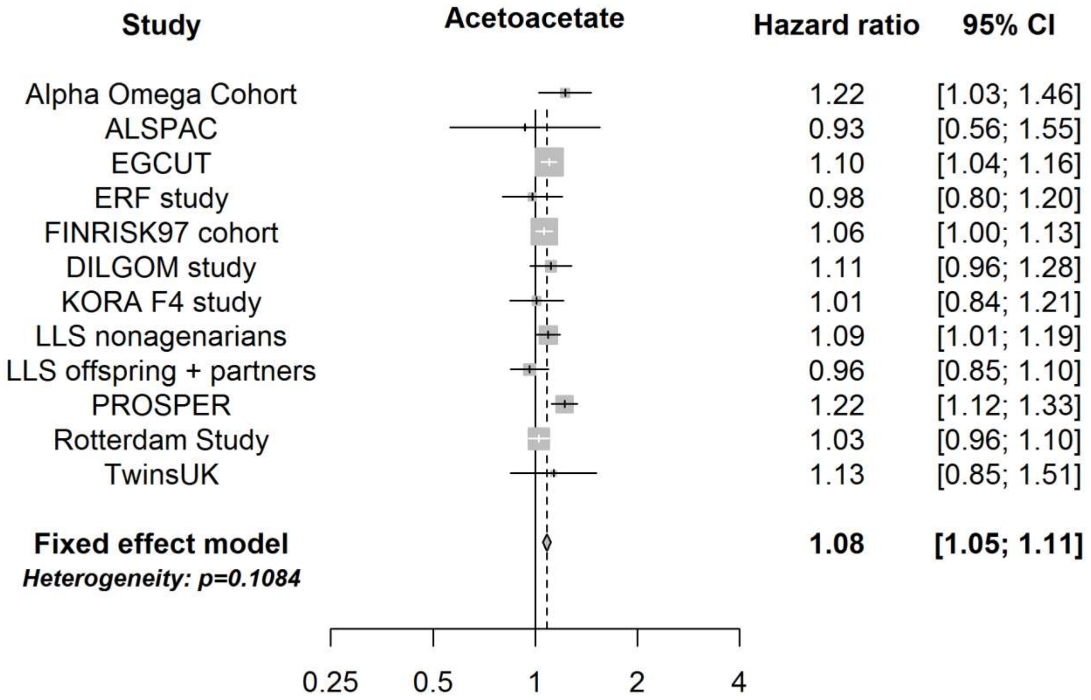

Supplementary Figure 13. Forest plot for acetoacetate based on the fully adjusted model.

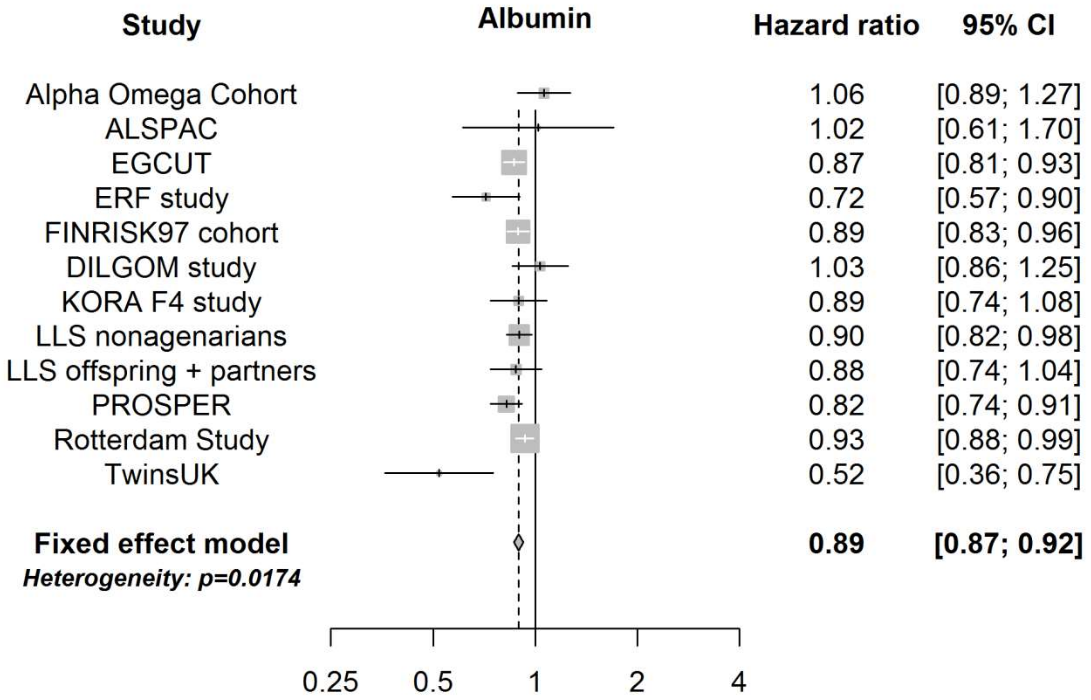

Supplementary Figure 14. Forest plot for albumin based on the fully adjusted model.

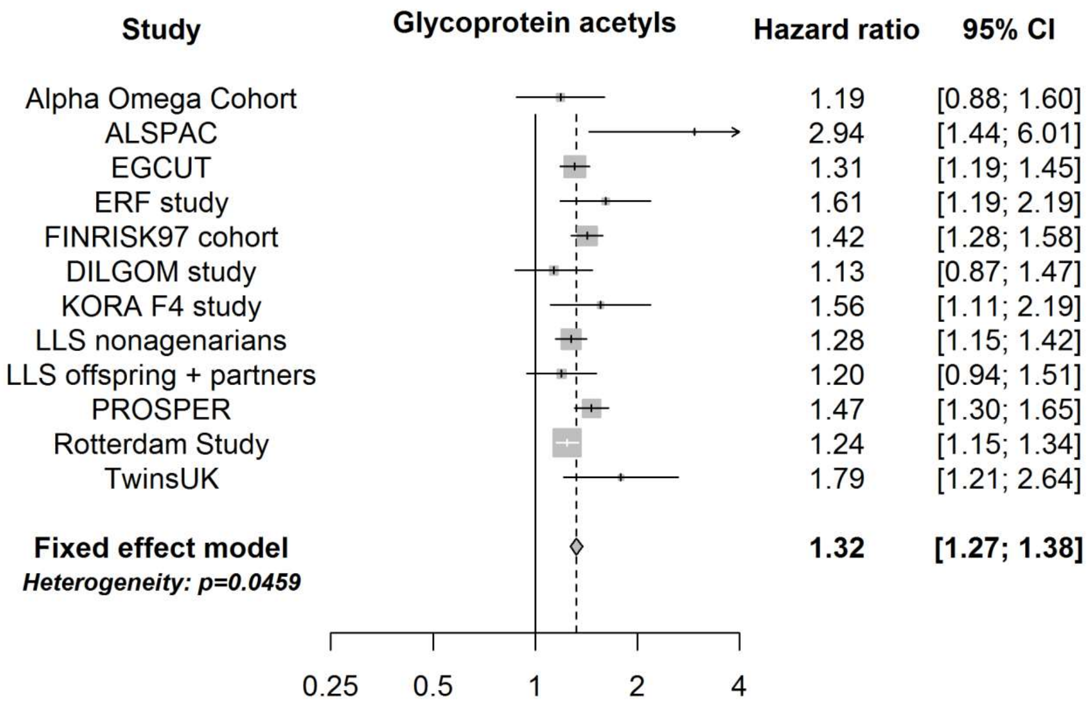

Supplementary Figure 15. Forest plot for glycoprotein acetyls based on the fully adjusted model.

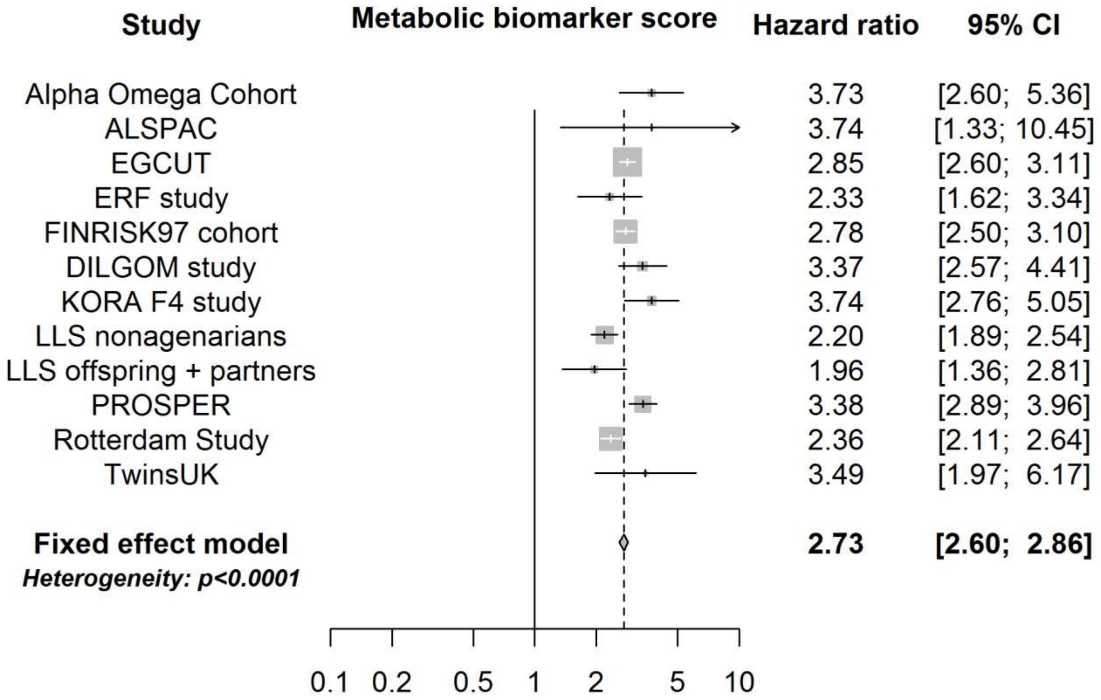

Supplementary Figure 16. Forest plot for the metabolic biomarker score based on the fully adjusted model.

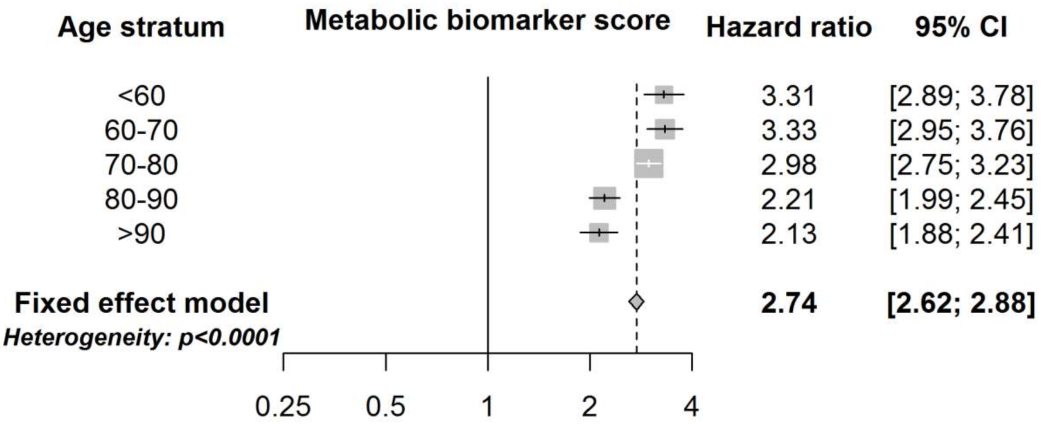

Supplementary Figure 17. Forest plot the metabolic biomarker score, stratified by age.

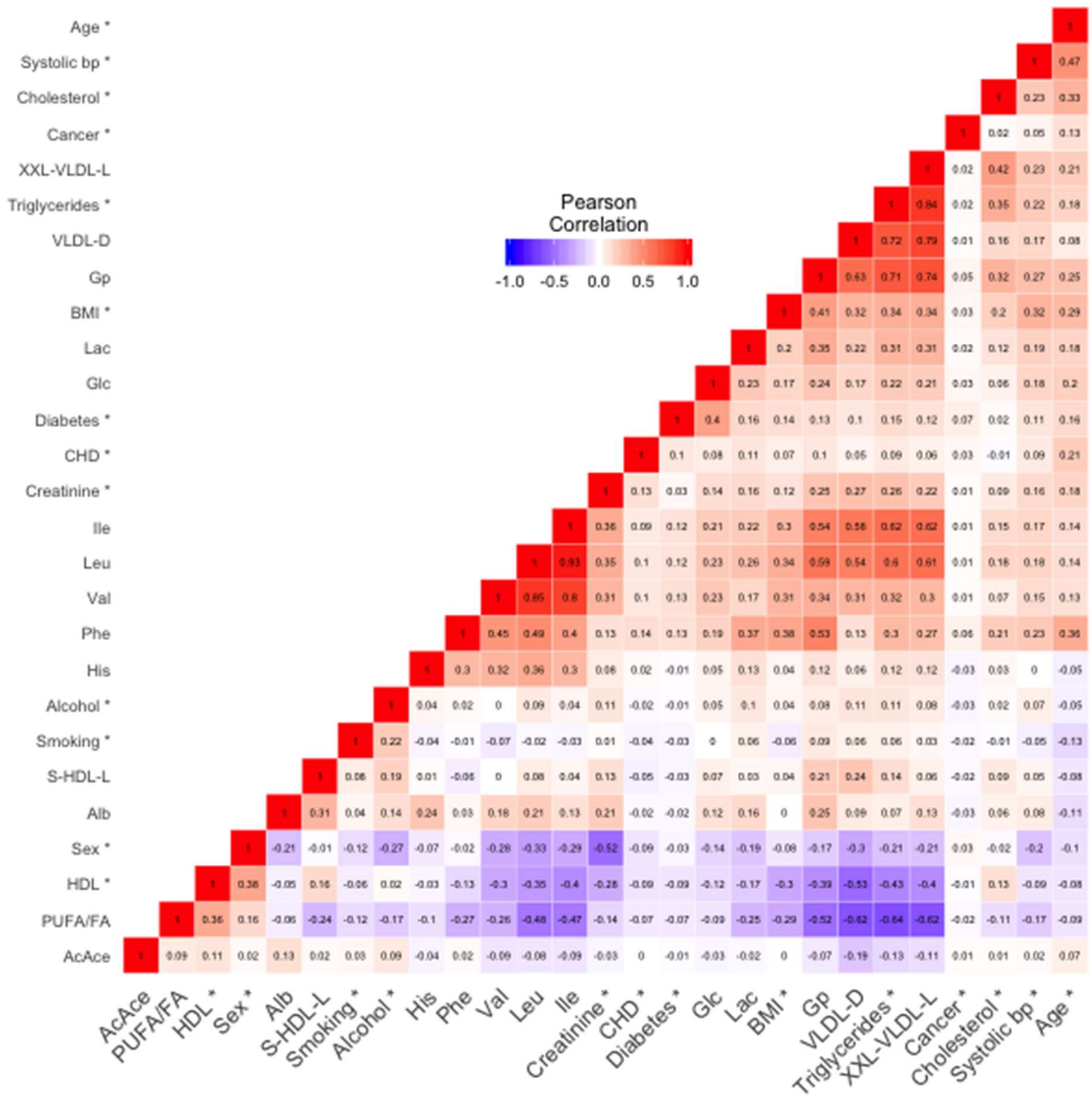

Supplementary Figure 18. Correlations between the conventional risk factors and 14 metabolic biomarkers. The conventional risk factors are depicted with an *.

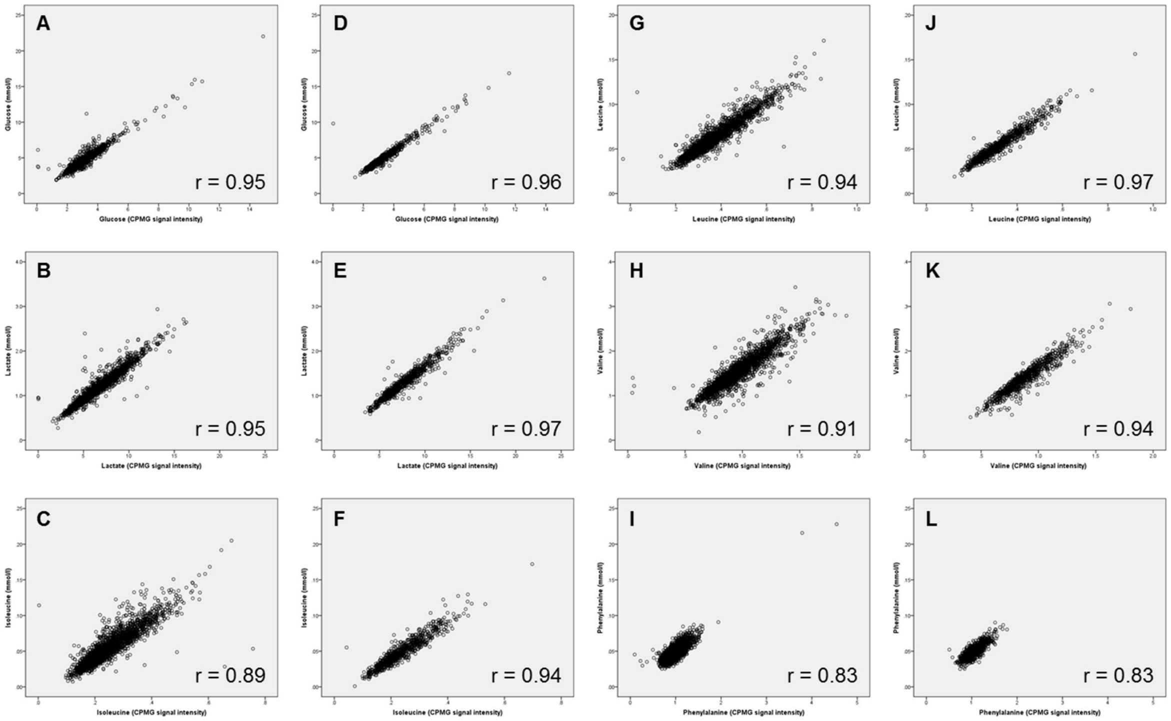

Supplementary Figure 19. Reproducibility of the quantification of some of the identified metabolic biomarkers. Comparison of the metabolic biomarkers glucose (A and D), lactate (B and E), isoleucine (C and F), leucine (G and J), valine (H and K) and phenylalanine (I and L) as measured with in-house NMR (x-axis) and the Nightingale Health platform (y-axis) in the Leiden Longevity Study offspring and partners (n = 2,164, A,B,C,G,H, and I) and nonagenarians (n = 802, D,E,F,J,K, and L).

Supplementary Tables

Supplementary Table 1. Results of the discrimination and reclassification analyses for all-cause mortality in the FINRISK 1997 cohort comparing the two metabolic biomarker scores.

Follow-up time	Age	Metabolic biomarker score Fischer C-statistic	Metabolic biomarker score C-statistic	Difference in C-statistic	IDI
5 years	All	0.811	0.837	0.026±0.011, P=0.017	1.9±1.3%, P=0.13
5 years	>60	0.685	0.732	0.048±0.019, P=0.013	2.0±1.7%, P=0.25
10 years	All	0.802	0.830	0.028±0.006, P=1.23 x 10^{-6}	2.3±0.8%, P=0.006
10 years	>60	0.667	0.715	0.048±0.011, P=2.34 x 10^{-5}	3.0±1.2%, P=0.018

The estimates for the risk scores were derived from the Estonian Biobank cohort. The *Metabolic biomarker score Fischer* included very low density lipoprotein (VLDL) diameter, albumin, glycoprotein acetyls (GlycA), citrate, and sex. The *Metabolic biomarker score* included total lipids in chylomicrons and extremely large VLDL, total lipids in small high-density lipoprotein, VLDL diameter, ratio of polyunsaturated fatty acids to total fatty acids, glucose, lactate, histidine, isoleucine, leucine, valine, phenylalanine, acetoacetate, albumin, GlycA, and sex. *IDI*; integrated discrimination improvement.

Supplementary Table 2. Results of the discrimination and reclassification analyses for all-cause mortality in the FINRISK 1997 cohort comparing the metabolic biomarker score with a score combining the biomarkers and conventional risk factors.

Follow-up time	Age	Metabolic biomarker score C-statistic	Combined score C-statistic	Difference in C-statistic	IDI
5 years	All	0.837	0.783	-0.05±0.018, P=0.002	-2.3±1.1%, P=0.03
5 years	>60	0.732	0.695	-0.038±0.021, P=0.07	-1.6±1.1%, P=0.16
10 years	All	0.830	0.783	-0.047±0.009, P=1.56 x 10^{-6}	-0.9±0.7%, P=0.23
10 years	>60	0.715	0.700	-0.015±0.011, P=0.19	-0.5±1.0%, P=0.58

The estimates for the risk scores were derived from the Estonian Biobank cohort. The *Metabolic biomarker score* included total lipids in chylomicrons and extremely large very low density lipoprotein (VLDL), total lipids in small high-density lipoprotein (HDL), VLDL diameter, ratio of polyunsaturated fatty acids to total fatty acids, glucose, lactate, histidine, isoleucine, leucine, valine, phenylalanine, acetoacetate, albumin, glycoprotein acetyls (GlycA), and sex. The *Combined score* included total lipids in chylomicrons and extremely large VLDL, total lipids in small HDL, VLDL diameter, ratio of polyunsaturated fatty acids to total fatty acids, glucose, lactate, histidine, isoleucine, leucine, valine, phenylalanine, acetoacetate, albumin, GlycA, sex, body mass index, systolic blood pressure, smoking status, alcohol consumption, and prevalent diabetes, cardiovascular disease, and cancer. *IDI*; integrated discrimination improvement.

Supplementary Table 3. Correlations of the identified metabolic biomarkers associated with mortality as measured with different commercially available metabolomics platforms.

Platform	**Metabolon**	**Biocrates p150**
Study	**FINRISK 1997 cohort**	**LLS offspring + partners**
Samples	**681**	**654**
Glc	0.82	0.85
Lac	0.76	NA
His	0.42	0.52
Ile	0.71	NA
Leu	0.66	0.78
Val	0.71	0.74
Phe	0.63	0.60

NA; not available.

Supplementary Table 4. Weights used for the risk scores tested in the FINRISK 1997 cohort (based on the Estonian Biobank cohort).

Parameter	Conventional risk factors	Metabolic biomarkers	Metabolic biomarkers Fischer et al.	Conventional risk factors + metabolic biomarkers
Systolic blood pressure	0.002			0.004
Body mass index	0.001			0.006
Total cholesterol	0.003			
High density lipoprotein cholesterol	-0.389			
Triglycerides	-0.098			
Creatinine	4.139			
Smoking status	0.942			0.734
Alcohol consumption	0.023			-0.016
Prevalent diabetes	0.519			0.355
Prevalent cardiovascular disease	0.247			0.202
Prevalent cancer	0.474			0.402
Sex	-0.402	-0.685	-0.776	-0.561
XXL-VLDL-L		0.022		0.032
S-HDL-L		-0.123		-0.141
VLDL-D		-0.245	-0.360	-0.290
PUFA/FA		-0.253		-0.222
Glc		0.246		0.175
Lac		0.130		0.092
His		-0.067		-0.061
Ile		0.056		0.083
Leu		-0.318		-0.256
Val		-0.035		-0.101
Phe		0.115		0.141
AcAce		0.100		0.081
Alb		-0.149	-0.384	-0.127
GlycA		0.272	0.525	0.246
Cit			0.289	

Supplementary References

1. Geleijnse JM*, et al.* Effect of low doses of n-3 fatty acids on cardiovascular diseases in 4,837 post-myocardial infarction patients: design and baseline characteristics of the Alpha Omega Trial. *Am Heart J* **159**, 539-546 e532 (2010).

2. Kromhout D, Giltay EJ, Geleijnse JM, Alpha Omega Trial G. n-3 fatty acids and cardiovascular events after myocardial infarction. *N Engl J Med* **363**, 2015-2026 (2010).

3. Fraser A*, et al.* Cohort Profile: the Avon Longitudinal Study of Parents and Children: ALSPAC mothers cohort. *Int J Epidemiol* **42**, 97-110 (2013).

4. Burton PR*, et al.* Data Safe Havens in health research and healthcare. *Bioinformatics* **31**, 3241-3248 (2015).

5. Pardo LM, MacKay I, Oostra B, van Duijn CM, Aulchenko YS. The effect of genetic drift in a young genetically isolated population. *Ann Hum Genet* **69**, 288-295 (2005).

6. Leitsalu L*, et al.* Cohort Profile: Estonian Biobank of the Estonian Genome Center, University of Tartu. *Int J Epidemiol* **44**, 1137-1147 (2015).

7. Vartiainen E*, et al.* Thirty-five-year trends in cardiovascular risk factors in Finland. *Int J Epidemiol* **39**, 504-518 (2010).

8. Konttinen H, Mannisto S, Sarlio-Lahteenkorva S, Silventoinen K, Haukkala A. Emotional eating, depressive symptoms and self-reported food consumption. A population-based study. *Appetite* **54**, 473-479 (2010).

9. Wichmann HE, Gieger C, Illig T, Group MKS. KORA-gen--resource for population genetics, controls and a broad spectrum of disease phenotypes. *Gesundheitswesen* **67 Suppl 1**, S26-30 (2005).

10. Schoenmaker M*, et al.* Evidence of genetic enrichment for exceptional survival using a family approach: the Leiden Longevity Study. *Eur J Hum Genet* **14**, 79-84 (2006).

11. Shepherd J*, et al.* The design of a prospective study of Pravastatin in the Elderly at Risk (PROSPER). PROSPER Study Group. PROspective Study of Pravastatin in the Elderly at Risk. *Am J Cardiol* **84**, 1192-1197 (1999).

12. Hofman A*, et al.* The Rotterdam Study: 2016 objectives and design update. *Eur J Epidemiol* **30**, 661-708 (2015).

13. Moayyeri A, Hammond CJ, Valdes AM, Spector TD. Cohort Profile: TwinsUK and healthy ageing twin study. *Int J Epidemiol* **42**, 76-85 (2013).
