# Supplementary material for: A metabolic profile of all-cause mortality risk identified in an observational study of 44,168 individuals
Source: Nat Commun. 2019 Aug 20;10:3346. doi: 10.1038/s41467-019-11311-9 (PMC6702196; doi:10.1038/s41467-019-11311-9)
Supplement: Supplementary file 3 — Description of Additional Supplementary Files [file 41467_2019_11311_MOESM3_ESM.pdf]

Description of Additional Supplementary Files

File name: Supplementary Data 1

Description: Association of metabolic biomarkers with all-cause mortality.

File name: Supplementary Data 2

Description: Description of NMR-measured metabolic biomarkers included in this study.

File name: Supplementary Data 3

Description: Association of metabolic biomarkers with all-cause mortality, stratified by sex.

File name: Supplementary Data 4

Description: Age-stratified association of metabolic biomarkers with all-cause mortality.

File name: Supplementary Data 5

Description: Script used for the scaling of the metabolic biomarkers of the Alpha Omega Cohort.

File name: Supplementary Data 6

Description: Script used for analyses of the Alpha Omega Cohort.

File name: Supplementary Data 7

Description: Script used for the meta-analyses.
